# Characterization of circulating transfer RNA-derived RNA fragments in cattle

**DOI:** 10.3389/fgene.2015.00271

**Published:** 2015-08-24

**Authors:** Eduardo Casas, Guohong Cai, John D. Neill

**Affiliations:** National Animal Disease Center, United States Department of Agriculture – Agricultural Research ServiceAmes, IA, USA

**Keywords:** cattle, RNA-seq, small non-coding RNA, tRF, transfer RNA fragments

## Abstract

The objective was to characterize naturally occurring circulating transfer RNA-derived RNA fragments (tRFs) in cattle^1^. Serum from eight clinically normal adult dairy cows was collected, and small non-coding RNAs were extracted immediately after collection and sequenced by Illumina MiSeq. Sequences aligned to transfer RNA (tRNA) genes or their flanking sequences were characterized. Sequences aligned to the beginning of 5′ end of the mature tRNA were classified as tRF5; those aligned to the 3′ end of mature tRNA were classified as tRF3; and those aligned to the beginning of the 3′ end flanking sequences were classified as tRF1. There were 3,190,962 sequences that mapped to transfer RNA and small non-coding RNAs in the bovine genome. Of these, 2,323,520 were identified as tRF5s, 562 were tRF3s, and 81 were tRF1s. There were 866,799 sequences identified as other small non-coding RNAs (microRNA, rRNA, snoRNA, etc.) and were excluded from the study. The tRF5s ranged from 28 to 40 nucleotides; and 98.7% ranged from 30 to 34 nucleotides in length. The tRFs with the greatest number of sequences were derived from tRNA of histidine, glutamic acid, lysine, glycine, and valine. There was no association between number of codons for each amino acid and number of tRFs in the samples. The reason for tRF5s being the most abundant can only be explained if these sequences are associated with function within the animal.

## Introduction

High throughput sequencing has allowed the identification of small non-coding RNA (Cole et al., 2009; Lee et al., 2009). Transfer RNA-derived RNA fragments (tRFs) are a component of small non-coding RNA (Haussecker et al., 2010). MicroRNAs (miRNA), small interfering RNA (siRNA), and Piwi-interfering RNA (piRNA), are also considered sncRNA. MicroRNAs have been the most studied because of their regulatory functions (Bartel, 2009; Stewart et al., 2013).

The tRFs were previously considered a degradation product; however, they were associated with regulation of translation in the cell (Cole et al., 2009; Garcia-Silva et al., 2012; Sobala and Hutvagner, 2013). It has been established tRFs are not randomly generated and have distinct size (Kumar et al., 2014). Their nomenclature is based on the processing site of the transfer RNA (tRNA): tRFs processed from the 5′ or 3′ end of the mature RNA are termed tRF5 and tRF3, respectively, while those produced from the 3′ sequence cleaved from the immature tRNA are designated tRF1 (Lee et al., 2009; Haussecker et al., 2010; Kumar et al., 2014). These RNAs are the second most abundant, after microRNAs (Lee et al., 2009).

It has been suggested that tRFs have similar function as microRNAs (Haussecker et al., 2010; Garcia-Silva et al., 2012). MicroRNA and tRFs are created by analogous processes in the cell, where the last step is its cleavage by the endonuclease Dicer (Garcia-Silva et al., 2012; Sobala and Hutvagner, 2013). Once cleaved by Dicer, tRFs are involved in RNA silencing through their association with Argonaut proteins.

It has been proposed that tRFs are produced in the bone marrow and in the immune cells (Nolte-’t Hoen et al., 2012), although other cells may also have the ability to produce them (Thompson and Parker, 2009; Ivanov et al., 2011). These molecules have been identified as being involved in inhibition of gene expression, in cell stress (Thompson and Parker, 2009), and in virus replication (Wang et al., 2013). Given that tRFs play a role in inhibiting gene expression or in virus replication, it is possible these functions may be involved in mediating infection-induced defense response by organisms (Zhang et al., 2014).

It is now known that tRFs are highly abundant molecules in different cell lines and tissues from human and mouse (Bartel, 2009; Kumar et al., 2014), and have also been identified in databases from plants (Loss-Morais et al., 2013). A single study has characterized tRFs associated with human cell lines challenged with respiratory syncytial virus (Wang et al., 2013), but no attempt has been made to characterize naturally occurring tRFs in homeostatic cattle, which is the objective of this study.

## Materials and Methods

### Animals

Serum from eight adult dairy cows was obtained from the dairy barn from the National Animal Disease Center, in Ames, IA, USA. Bleeding of animals was done according to the management protocol approved by the Animal Care and Use Committee of the Institution. Fresh serum was obtained from eight adult female Holstein cows by jugular venipuncture in SST vacutainer tubes (BD, Franklin Lakes, NJ, USA). The vacutainer tubes were incubated at 37°C degrees for 30 min and were centrifuged at 1250 × g for 30 min. The isolated serum samples were placed into 2 ml vials and were stored at -80°C until processed.

### tRF Isolation

The tRFs were isolated from the serum samples using the miRNeasy Serum/Plasma kit (QIAGEN, Germantown, MD, USA) using 200 ul of serum sample. The tRFs was extracted according to manufactures direction and the samples were eluted in 14 ul of RNase free water. After extraction 1 ul of each sample were run using the Small RNA chip on an Agilent 2100 Bioanalyzer (Agilent Technologies, Santa Clara, CA, USA) to quantify the serum extracted from the samples. The tRFs concentration was determined by using a 10–40 nucleotide gate.

### Library Preparation

Each extracted serum sample was used to prepare sequencing libraries. The libraries were prepared using the NEBNext Multiplex Small RNA Library Prep Set for Illumina Set 1 and 2 (New England BioLabs, Ipswich, MA, USA). The libraries were individually index with the Illumina 1–24 indexed primers. Six microliters (6 ul) of each animal’s isolated serum was used in library preparation according to manufacturer’s instructions. After the library preparation the libraries were cleaned up and concentrated using the QIAquick PCR purification kit (QIAGEN, Germantown, MD, USA) from 100 to 27.5 ul. The quality and quantity of the libraries were determined by running 1 ul of each library on a DNA 1000 chip on an Agilent 2100 Bioanalyzer (Agilent Technologies, Santa Clara, CA, USA). The concentration of each indexed library was determined by using a 135–170 nucleotide gate. All the indexed libraries were then pooled and size selected. Five nanograms of each indexed library used to make the pool. The total volume of the pool was 246.5 ul. The pool was concentrated using the QIAquick PCR purification kit (QIAGEN, Germantown, MD, USA) to 35 ul of RNase free water. The pool was then size selected using the Pippin Prep on a 3% Agarose gel without added Ethidium Bromide (SAGE Sciences, Beverly, MA, USA) with a size selection of 142–170 nt according to manufactures instructions. After the gel was run the pools were concentrated using the QIAquick PCR purification kit (QIAGEN, Germantown, MD, USA) by eluting in 32 ul of RNase free water. One microliter of the size selected library pool was run using a High sensitivity DNA chip Agilent 2100 Bioanalyzer (Agilent Technologies, Santa Clara, CA, USA). The concentration was determined by using a 135–170 nucleotide gate. The final concentration of the size selected pool library was 1.5 nM and the pool was stored at -20°C.

### Sequencing the Library Pool

The pooled size selected library was sequenced on a MiSeq using the MiSeq Sequencing Kit v2 50 Cycles (Illumina, San Diego, CA, USA) in the Sequencing Core Facility at the National Animal Disease Center (NADC).

### Data Analysis

The quality of Illumina sequences was inspected using FastQC v0.11.2^2^. The Illumina adapter was removed using fastx_clipper program in the fastx toolkit^3^. Multiple occurrences of unique reads were merged using a custom script. Reads 18–40 bp were used in downstream analysis. They were first mapped to *Bos taurus* genome (ENSEMBL UMD3.1.75) using Novoalign software (Novocraft Technologies) allowing two mismatches (-o SAM –o FullNW –g 99 –r Random –t 60). Those aligned to the genome were then mapped to a database containing different annotated genome features to determine their origin (-o SAM –o FullNW –g 99 –r All –t 60): genomic tRNA sequences were downloaded from http://gtrnadb.ucsc.edu/; mitochondrial tRNA, cDNA, and other non-coding RNA sequences were downloaded from ENSEMBL version 75. To account for tRFs derived from tRNA precursors, such as tRF1, 40-bp flanking sequences on both ends of tRNA genes were retrieved from the genome and appended to the tRNA genes. The Illumina reads aligned to tRNA genes or their flanking sequences were further characterized. They were aligned to *Bos taurus* tRNA database using BLASTN and the results were processed using a custom script. Those perfectly aligned to the beginning of mature tRNAs were classified as tRF5; those perfectly aligned to the end of mature tRNA, with or without the CCA sequences at the 3′ end, were classified as tRF3; and those aligned to the beginning of the 3′ end flanking sequences without mismatch were classified as tRF1. After tRF5, tRF3, and tRF1 sequences have been determined, their occurrences in Illumina sequences from individual animals were obtained using a custom script. Sequences have been submitted to NCBI Short Read Archive^4^, under BioProject accession PRJNA289352.

All sequences were obtained from sera, and similar tRF composition and count of sequences were expected for all samples. Sequences were normalized using the following methodology:

Normalized Counts = Raw Counts ^∗^ mean library size/library size of individual sample.

Statistical correlations were determined between normalized tRF5s and number of codons per amino acid, and between normalized tRF5s and number of genes, using the PROC CORR procedure from SAS (SAS Inst. Inc., Cary, NC, USA).

## Results

There were a total of 5,078,907 sequences obtained in the present study. From these, 4,466,139 sequences were between 18 and 40 nucleotides in length. There were 3,190,962 sequences that mapped to tRNA and small non-coding RNAs in the bovine genome. Of these, 2,323,520 were identified as tRF5s, 562 were tRF3s, and 81 were tRF1s. There were 866,799 sequences identified as small non-coding RNAs (microRNA, rRNA, snoRNA, etc.) and were excluded from the study.

For identification purposes, the nomenclature of tRFs will be according to the tRNA of origin. The number of tRF5 sequences by size is shown in **Table 1**. The tRF5s ranged from 28 to 40 nucleotides. There were fewer than 2,000 sequences for each tRF5 ranging from 15 to 18 nucleotides in length, and fewer than 4,000 sequences for each tRF5 ranging from 19 to 27 nucleotides in length. Each sequence ranging from 15 to 27 nucleotides in length represented less than 0.2% of all the sequences and were removed from the study. Sequences ranging from 30 to 34 nucleotides in length comprised 98.7% of all sequences. The largest number of sequences was observed at 31 nucleotides (27.6%), followed by 34 nucleotides (25.9%). These two nucleotide sizes account for 53.5% of the total number of sequences. The most abundant tRF5s were tRF5-His, tRF-Glu, tRF5-Lys, tRF5-Gly, and tRF5-Val. For tRF5-His, the majority were 34 nucleotides in length, followed by a population of 31 nucleotides. For tRF5-Glu, the largest number was 31 nucleotides, followed by the second most abundant of 33 nucleotides. For tRF5-Gly, the most common was 30 nucleotides in length, followed by 31 nucleotides. For tRF5-Lys and tRF5-Val, the most common was 32 nucleotides in length, followed by 31 nucleotides in length. Regardless of the tRF5, in all cases, the length with the most number of sequences had more than double the amount when compared to the second most common.

**Table 1:** Number of transfer RNA derived RNA fragments (tRF5) according to length in nucleotides (nt).

	Length (nt)													
tRF5	28	29	30	31	32	33	34	35	36	37	38	39	40	Total
tRF5-Ala	32	584	2304	387	172	1	0	0	0	0	0	0	0	3480
tRF5-Arg	0	0	0	1	0	3	0	0	0	0	0	0	0	4
tRF5-Asn	0	0	1	0	0	0	0	0	0	0	0	0	0	1
tRF5-Asp	0	0	0	0	0	5	46	8	3	0	0	0	0	62
tRF5-Cys	4	3	5	45	169	54	3	2	0	0	0	0	0	285
tRF5-Gln	27	29	1381	1044	10	79	105	11	7	0	0	0	5	2698
tRF5-Glu	761	5235	45495	356687	112717	134146	22158	5165	916	41	58	1291	1047	685717
tRF5-Gly	2811	7191	311707	64428	34303	969	203	144	545	3	6	1	71	422382
tRF5-His	38	454	72659	119840	76384	8686	553427	27	10	0	0	0	2	831527
tRF5-Ile	0	0	0	0	0	0	0	0	0	0	0	0	0	0
tRF5-Leu	0	0	0	0	1	3	13	0	0	0	0	0	0	17
tRF5-Lys	454	37	1158	16529	26139	3400	895	50	29	2	1	6	69	48769
tRF5-Met	0	0	8	56	0	0	0	0	0	0	0	0	0	64
tRF5-Phe	0	0	0	0	0	0	1	0	0	0	0	0	0	1
tRF5-Pro	286	301	1250	2295	1001	1047	4	2	0	0	0	0	0	6189
tRF5-Ser	0	0	0	0	2	0	0	0	0	0	0	0	0	2
tRF5-Stop	0	1	0	0	0	0	7	391	26	13	0	0	0	438
tRF5-Thr	0	1	19	49	44	0	0	0	0	0	0	0	0	113
tRF5-Trp	0	0	0	0	0	0	0	0	0	0	0	0	0	0
tRF5-Tyr	0	0	0	0	0	0	0	0	0	0	0	0	0	0
tRF5-Val	59	888	1321	80220	160655	54321	24171	30	53	40	3	8	5	321774
**Total**	4472	14724	437308	641581	411597	202714	601033	5830	1589	99	68	1306	1199	2323520
**Proportion**	0.002	0.006	0.188	0.276	0.177	0.087	0.259	0.002	0.001	0.00	0.00	0.001	0.001	

There are several patterns regarding length of tRF5 (**Table 1**). tRF5-His had the largest number of sequences and most ranged from 28 to 36 nucleotides in length. The tRF5 for tRF5-Glu, tRF5-Gly, tRF5-Lys, and tRF5-Val, ranged from 28 to 40 nucleotides. Although tRF5-Ala had more than 1,000 sequences, the length ranged from 28 to 33 nucleotides in length. The length of tRF5-Gln ranged from 28 to 36 nucleotides.

The number of tRF5s for each animal in the study is shown on **Table 2**. There were more than 2.3 million tRF5s, from approximately 5.1 million total sequences (46% of the total sequences). The number of tRF5s for each animal ranged from 259,480 to 316,580, with an average of 290,440. The number of tRF5s identified for each animal is consistent across all animals, without any given animal having extreme numbers of sequences.

**Table 2:** Number of normalized 5′ transfer RNA derived RNA fragments (tRF5) in each animal in the study.

	Animal ID								Total tRFs	tRFs/cow	tRNA genes
tRF5	1141	1143	2919	2927	2931	2938	2939	2942			
tRF5-Ala	544	352	408	518	351	362	479	419	3433	429	83
tRF5-Arg	2	0	1	0	0	1	0	0	4	0.5	204
tRF5-Asn	0	0	0	0	0	0	1	0	1	0.125	40
tRF5-Asp	12	5	18	4	9	8	6	2	64	8	54
tRF5-Cys	27	21	30	94	9	43	27	25	276	35	510
tRF5-Gln	152	432	313	359	394	307	455	307	2719	340	60
tRF5-Glu	93648	88823	108094	78000	106609	78579	66925	71478	692156	86520	621
tRF5-Gly	41814	56821	61251	60975	45043	60536	46838	49574	422852	52857	1795
tRF5-His	75542	133863	116715	73717	140707	96272	111728	96764	845308	105664	25
tRF5-Ile	0	0	0	0	0	0	0	0	0	0	25
tRF5-Leu	3	1	4	5	1	1	2	0	17	2	49
tRF5-Lys	5635	8000	6634	5972	5206	6484	6237	4596	48764	6096	121
tRF5-Met	5	8	11	14	7	6	8	4	63	8	33
tRF5-Phe	1	0	0	0	0	0	0	0	1	0.125	35
tRF5-Pro	512	505	554	646	781	666	1277	1225	6166	771	26
tRF5-Ser	0	0	1	1	0	0	0	0	2	0.25	79
tRF5-Stop	94	25	94	28	35	71	51	38	436	55	25
tRF5-Thr	24	13	14	8	11	15	18	8	111	14	132
tRF5-Trp	0	0	0	0	0	0	0	0	0	0	182
tRF5-Tyr	0	0	0	0	0	0	0	0	0	0	48
tRF5-Val	34225	37307	44317	40587	31800	40946	54467	36366	320015	40002	90
**Total tRF5**	252240	326176	338459	260928	330963	284297	288519	260806	2342388	292799	

In **Table 2** it was observed there were five populations of tRF5 with more than 1000 per animal (tRF5-Glu, tRF5-Gly, tRF5-His, tRF5-Lys, and tRF5-Val). tRF5-His had the largest number per animal, followed by tRF5-Glu, tRF5-Gly, tRF5-Val, and tRF5-Lys. Three tRF5s, tRF5-Ala, tRF5-Gln, and tRF5-Pro, had more than 100 and fewer than 1000 sequences per animal. Two tRF5s, tRF5-Trp and tRF5-Tyr, had no sequences. The remaining tRF5s had at least 1, but fewer than 100 sequences. The number of anticodons for each amino acid range from 1 to 6 for different tRNAs. There was no correlation between number of anticodons per amino acid and number of tRF5.

**Table 2** shows the number of genes in the bovine genome for each tRF. tRF5-His had the largest number per animal, but there are only 25 genes for the tRNA that produced it. Opposite to tRF5-His, tRF5-Gly had the largest number of genes in the genome (*n* = 1,795), and was the third most abundant. tRF5-Glu was the second most abundant, and it had 621 tRNA genes in the bovine genome. tRF5-Val and tRF5-Lys were the fourth and fifth in abundance, there were only 90 and 121 tRNA genes in the bovine genome.

Correlations between number of codons per amino acid, number of genes, and count of normalized tRF5s was determined. The correlation between number of codons and normalized tRF5s was *r* = -0.12 (*P* = 0.59), and the correlation between number of genes and tRF5s was 0.39 (*P* = 0.08). Neither correlation was statistically significant; however, the latter correlation showed a tendency.

**Table 3** shows the number of tRF3s. There were 562 tRF3s in the animals under study. The number of tRF3s ranged from 41 to 107, with an average of 70 tRF3s per animal. The number of sequences was fewer than 100 in all cases. No sequences were observed for tRF3-His, tRF3-Ser, and tRF3-Stop. There were only three tRF1s identified in the study. Of the 81 sequences, 2 were tRF1-His, 76 were mitochondrial tRF1-Val, and 3 were mitochondrial tRF1-Tyr.

**Table 3:** Number of 3′ transfer RNA derived RNA Fragments (tRF3) in each animal in the study.

	Animal ID								Total tRFs	tRFs/cow
tRF3	1141	1143	2919	2927	2931	2938	2939	2942		
tRF3-Ala	1	0	1	2	1	0	0	0	5	0.625
tRF3-Arg	1	4	4	10	1	7	2	4	33	4.125
tRF3-Asn	13	6	18	8	3	6	14	6	74	9.250
tRF3-Asp	6	3	9	6	3	3	6	4	40	5
tRF3-Cys	0	1	0	3	0	0	0	0	4	0.500
tRF3-Gln	2	1	1	2	1	1	0	1	9	1.125
tRF3-Glu	34	16	41	43	25	26	24	46	255	31.875
tRF3-Gly	0	0	1	4	0	1	2	0	8	1
tRF3-His	0	0	0	0	0	0	0	0	0	0
tRF3-Ile	0	1	0	0	0	0	0	0	1	0.125
tRF3-Leu	1	0	0	0	1	0	2	2	6	0.750
tRF3-Lys	4	2	7	2	0	5	4	12	36	4.500
tRF3-Met	3	2	1	1	1	1	0	0	9	1.125
tRF3-Phe	0	1	0	0	0	0	1	0	2	0.25
tRF3-Pro	0	1	1	3	0	0	0	0	5	0.625
tRF3-Ser	0	0	0	0	0	0	0	0	0	0
tRF3-Stop	0	0	0	0	0	0	0	0	0	0
tRF3-Thr	1	0	3	7	1	3	2	1	18	2.250
tRF3-Trp	2	0	1	0	1	0	1	0	5	0.625
tRF3-Tyr	0	1	2	0	0	1	1	0	5	0.625
tRF3-Val	6	2	5	16	3	7	2	6	47	5.875
**Total tRF3**	47	41	95	107	41	61	61	82	562	

## Discussion

It has been hypothesized that tRFs may be a significant regulator of gene silencing when animals are faced with a pathogen that may modify their homeostatic status, causing disease (Wang et al., 2013). A limited number of studies have evaluated tRFs in cell lines, mice, and plants (Cole et al., 2009; Loss-Morais et al., 2013; Maute et al., 2013; Kumar et al., 2014), and only one study has evaluated tRFs response to a bovine virus in bovine cell culture (Wang et al., 2013). The present study provides an overall characterization of tRFs that are circulating in cattle. This information is necessary to understand difference in expression profiles when pathogen challenge studies in live animals are conducted.

Production of tRFs has been observed when the cell is under stress (Zhang et al., 2014). The stress can be physical, chemical, or by a viral infection (Thompson and Parker, 2009; Wang et al., 2013). Zhang et al. (2014), using mice as an animal model, observed that during an acute inflammation process, there was an increase of serum tRFs during the first days of the inflammation, followed by a decrease a few days afterward. An initial screening of human patients with an active Hepatitis B virus infection also revealed an increase in serum tRFs (Zhang et al., 2014). Zhang et al. (2014) indicate that the biological significance and diagnostic value of serum tRFs warrants further studies and possibly opens a new round of research focused on serum tRFs. The objective of the present study was to characterize circulating tRFs in non-stressed bovine, with the ultimate goal to establish differences in tRF expression associated with diseases.

The tRFs have been classified according to their cleavage portion of tRNA present (Lee et al., 2009). Their production and role within the cell has been studied, but no indication of their relative abundance has been established in humans, animals, or plants (Garcia-Silva et al., 2012; Loss-Morais et al., 2013; Sobala and Hutvagner, 2013; Kumar et al., 2014). In the present study we have determined that under normal conditions, tRF5s are predominant, while tRF3 and tRF1 are present at a very low level in cattle sera. Wang et al. (2013), indicated that high throughput sequencing data captured tRF5, tRF3, and tRF1 in comparable quantities in prostate cancer cells. Increased production of tRF3 and tRF1 could be signs of changes in the homeostatic condition (i.e., disease), although further studies would be needed to establish this relationship.

The largest proportion of tRF5s was between 30 and 34 nucleotides in length, with tRF5s of 31 and 34 nucleotides being the most prevalent. Previous studies indicated that length of tRF5s was between 18 and 25 nucleotides (Lee et al., 2009) or between 21 and 22 nucleotides (Haussecker et al., 2010). Wang et al. (2013), identified a 31 nucleotide tRF5 (tRF5-Glu) as being a suppressor in messenger RNA in cytoplasm in the presence of bovine respiratory syncytial virus. It is unknown if differences in length are due to the location of the tRF5 (intra- or extra-cellular), if there are due to species difference in processing site, or are due to their function.

For each tRF5, the most abundant is almost double the amount of the next most abundant. As example, the most abundant tRF5-his was 34 nucleotides (*n* = 553,427), while the second most abundant was present at *n* = 119,840 (**Table 1**). This longest tRF5-his was produced 4.6 times greater than the second most common at 31 nucleotides. It will be necessary to further evaluate the function of each size of tRF5s to establish a comprehensive understanding of their function.

The association of number of tRNA genes in the genome, with tRF5s suggests that function rather than number is involved in production of tRFs. The most abundant tRF5s have a varying number of tRNA genes in the genome. The correlation between abundance of tRF5s and number of tRNA genes suggest that factors other than number of tRNA genes are influencing the production of tRF5s.

The sequence of each tRF is associated with a specific amino acid codon from which it originated. Function, rather than number of codons, should be associated with production of tRF5s. The tRF5-Glu identified by Wang et al. (2013) as being responsible for repressing mRNA in the cytoplasm is one of the most common in the present study. This is an indication that function drives the production of tRF5s. If the number of codons was to be responsible for production of tRF5s, amino acids with six codons should produce the largest amounts of tRF5s (tRF5-Arg, tRF5-Leu, and tRF5-Ser), which was not observed in the present study. Histidine, glutamine, glutamic acid, and lysine have two codons each, and the largest amounts of tRF5s were observed for their tRNAs. However, other amino acids with one or two codons (aspartic acid, aspargine, cysteine, phenylalanine, tryptophan, and tyrosine), had limited amounts of tRF5s. From amino acids with four codons, glycine and valine were the amino acids with the most tRF5s. However, other amino acids with four codons produced moderate numbers of tRF5s. The correlation between number of codons and amount of tRF5s produced by each amino acid is inconclusive. The reason why amino acids with the least number of codons have the most amounts of tRF5s can only be explained if these molecules are associated with function within the organism.

There is no association in production of tRF5s, tRF3s, and tRF1s. When Lee et al. (2009) described tRFs, they were able to clone and sequence equivalent number of sequences from three tRF families. Similarly, Sobala and Hutvagner (2013) and Kumar et al. (2014) observed similar proportions of the three tRFs families. The previously mentioned studies were done from cells grown *in vitro*. Production of tRFs in live organisms may be different, evidenced by the results of the present study and may not necessarily reflect what takes place in the animal.

There were several tRF5s with few sequences. The depth of coverage was adequate for the needs of the study according to Haas et al. (2012). Sequencing error rates should have occurred in the present study (Wang et al., 2012); however, there was no attempt to eliminate those tRFs with few sequences (de Sá et al., 2015). tRFs with low number of sequences could potentially be spurious results and should be taken into account when interpreting results from this study.

The largest number of sequences observed in the present study ranged from 29 to 35 nucleotides in length. Sequences ranging from 15 to 27 nucleotides in length were removed from the study due to their low frequency. Kumar et al. (2014) reported tRFs ranging from 14 to 32 nucleotides in length. Kumar et al. (2014) analyzed sequences from the NCBI SRA database and mapping them to the human, mouse, *Drosophila, Caenorhabditis elegans, Saccharomyces cerevisiae*, and *Schizosaccharomyces pombe*, while the present study comprised cattle sequences. It is possible that length of tRF5s is associated with the species.

Identification of tRFs is a relatively new field which is expanding due to the availability of high throughput sequencing. This is also the case for livestock production and health. We are embarking in identification of small non-coding RNA studies to establish their association with animals affected with diseases. The results from the present study could potentially be used as reference in cattle.

## Author Contributions

EC, GC, and JN conceived the project and interpreted results. EC and GC performed the experiment. EC wrote, and GC and JN reviewed the manuscript.

## Conflict of Interest Statement

Eduardo Casas, Guohong Cai, and John D. Neill are USDA employees and declare no conflict of interest.

## Footnotes

2 http://www.bioinformatics.babraham.ac.uk/projects/fastqc/

3 http://hannonlab.cshl.edu/fastx_toolkit/

4 http://www.ncbi.nlm.nih.gov/genbank/
